# The Perception and Experience of Parents of Children with Cleft Lip and Palate Concerning the Use Pre-Surgical Infant Orthopedics: A Questionnaire-Based Cross-Sectional Survey

**DOI:** 10.3390/children9071054

**Published:** 2022-07-15

**Authors:** Shaymaa Hadi Albustani, Arkadiusz Dziedzic, Mushriq Abid

**Affiliations:** 1 Department of Conservative Dentistry with Endodontics, University of Al-Qadisiyah, Al-Qadisiyah 13261, Iraq; shaymaa.albustani@qu.edu.iq; 2 Department of Restorative Dentistry with Endodontics, Medical University of Silesia, 40-055 Katowice, Poland; 3 Orthodontic Department, College of Dentistry, University of Baghdad, Baghdad 10071, Iraq

**Keywords:** perception, experience, attitude, cleft lip and palate, pre-surgical infant orthopedics

## Abstract

Background: A transitory period prior to the surgical correction of cleft lip and palate (CLP) is associated with adverse impacts, which may require a medical intervention. Pre-surgical infant orthopedics (PSIO) is deemed to reduce the functional and psychological burden, offering a transition until the definite surgical intervention. Aim: To assess the attitude of Iraqi mothers concerning the application and management of pre-surgical orthopedic appliances in children with cleft lip and palate, taking consideration of the mothers’ occupational status. Methods: The cross-sectional study was conducted in the College of Dentistry at Baghdad University from 5 January 2020 to 4 December 2021. A questionnaire form was validated based on existing data related to the assessment of parents’ satisfaction concerning PSIO. Results: A concern related to the impression procedure was reported by a minority of the participants (6.8%) and it was associated mainly to the perceptions of housewives’ vs. working mothers (*p* < 0.05). The perceived infants’ discomfort during the impression procedure reported at 11.9% was significantly associated with housewives’ status (*p* < 0.05). In general, the vast majority of respondents considered the impression as being non-invasive (96.6%). Most mothers found no difficulties in following the instructions of the specialist regarding the insertion of the PSIO and/or taping the elastic bands (62.7%). Respondents believed that CLP infants routinely require PSIO treatment. Interestingly, only a minority of mothers performed an Internet search to look for information about PSIO (7%). The majority indicated the PSIO treatment as beneficial for their infant and a substantial proportion of respondents were satisfied with the outcomes of PSIO, encouraging other parents to consent the PSIO treatment. Conclusion: In general, mothers broadly acknowledged the primary concept of PSIO and accepted the proposed treatment, with a positive attitude towards pre-surgical CLP management, regardless their socio-economic status. They seemed to understand well the expected benefits of PSIO, including feeding improvement, normalization of speech, and optimization of future surgical outcomes.

## 1. Introduction

One of the most common congenital abnormalities affecting the craniofacial region is cleft lip and palate, with a reported incidence in between 1:600 and 1:700 live births [1]. Children born with CLP frequently have feeding and psychological problems, impaired craniofacial development, and speech and common dental anomalies, such as hypodontia, supernumerary teeth, tooth shape and size malformations, as well as enamel defects [2,3]. The main objectives of the CLP management that starts early in infancy include feeding and aesthetic improvement, which are primarily focused on increasing columellar length, alveolar segment alignment, nostril symmetry achievement, and lip segment approximation [4,5]. Therefore, interdisciplinary team efforts, with orthodontist support, are crucial to provide optimized multi-staged treatment. A specialist cleft team should provide detailed oral health advice from six months of age. The timeline for cleft lip repair varies, and is usually between three and six months, whereas for cleft palate repair is 6–9 months [6]. An early intervention includes a combined primary and secondary/tertiary multidisciplinary care provision [7]. It has been suggested that the use of infant orthopedics before definite surgical correction can considerably facilitate these objectives in a favorable way [8]. As the quality of life of children with CLP anomaly is significantly decreased [9], caregivers/parents are often affected emotionally because of the complexity of the situation, including the available treatment the child must face during the first year of life. While a surgical correction is considered as the standard mode of treatment, a substantial proportion of children with CLP receives pre-surgical interventions aimed at reducing suffering in feeding and reducing the size of the defect [9].

**Figure 1:**
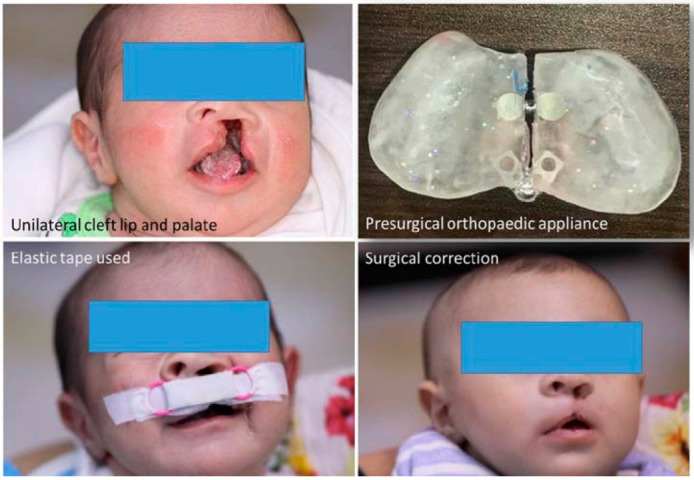
PSIO protocol in a CLP child and surgical treatment outcome.

The use of removable ortho-prosthetic appliances has been linked to several advantages, including the narrowing of the cleft lip and subsequently facilitating the surgical correction and the function of the tongue tip being more normalized [10]. Moreover, reducing feeding difficulties, the symmetry of the nose and maxilla being restored, ensuring the straight alignment of the nasal septum, enhanced speech evolution, and decreasing the deformities of skeleton and dentition are the primary benefits of the utilization of PSIO [11]. An example of PSIO is demonstrated on Figure 1.

Reviewing the available evidence-based data, the types of pre-surgical infant orthopedic appliances comprise active plates [12], passive plates [13], nasoalveolar molding [5], and pin-retained Latham’s appliance [14]. In the late 1970s, the intraoral plate was used to mold the maxillary segments prior to surgical repair reported by Hotz et al. [15]. Reportedly, the nasoalveolar molding was introduced later by Grayson et al. [5], providing a better approach toward achieving a clinical improvement prior to surgical goals. A lack of clinical and therapeutic consensus appears in the published literature regarding the use of pre-operative molding appliances. Currently, there is a strong acceptance of PSIO utilization [16,17,18,19]; however, controversies exist towards the long-term benefits of such CLP therapeutic approach [20,21,22,23]. Several systematic reviews have shown that nasoalveolar molding provides optimized therapeutic results as pre-surgical infant orthopedics [24,25,26,27].

The parents’ positive commitment and attitude are the principal bases upon which the success of pre-surgical appliances relies [28]. While the satisfaction of the parents of CLP children regarding PSIO acceptance has been studied in numerous cross-sectional survey-based studies [29,30,31,32,33,34,35], their aims were specifically dedicated toward the load on caregivers, while practicing the procedures of taking care of infants during the pre-operative period. Although an increasing burden has been reported by some researchers [33,34], other authors have stated opposite results [9,32]. It must be noted that selected research teams failed to infer a clear conclusion about the impact of using molding appliances [29,31]. In general, the burden and satisfaction expressed by caregivers with respect to the handling of nasoalveolar molding itself has not been evaluated. In addition, some authors did not specify the type of pre-surgical appliances, and equally did not focus on the satisfaction of parents as a primary outcome [24,27]. The several disadvantages of PSIO have been described, with the most important including its relatively high cost, delaying the time of surgical repair and drawing attention to the child’s deformity [14]. The introduction of removable palatal appliances dates back to several decades ago [36] and various types of appliances with different clinical indications, such as improving the arch form, aesthetic outcome, and nasal asymmetry, have been implemented in CLP management [37,38].

Due to the lack of consensus, unvalidated evidence-based data, and existing uncertainness with respect to parents’ satisfaction concerning the use of PSIO, the study aims to explore the attitudes of mothers with different occupational status regarding the application of pre-surgical orthopedic appliances in children with CLP. The ‘null hypothesis’ was evaluated comparatively using statistical tests.

## 2. Materials and Methods

The study was approved by the ethical committee of the College of Dentistry, University of Baghdad (reference number 262, project 262421). The cross-sectional survey-based study was conducted in the CLP academic center in the College of Dentistry at Baghdad University, as well as in two other dental clinics in Baghdad, lasting from 5 January 2020 to 4 December 2021. A sample size calculation was carried out using the following formula: N = N/1 + Z2 × P (1 − P)/E^2^N, where N: population size, Z: z score for % confidence interval, E: margin of error, and P: population proportion (0.5). A pre-tested and validated questionnaire form was originally prepared and applied to evaluate of the parents’ satisfaction towards PSIO. Parents were invited to complete self-administrated questionnaire that included information about the age of the infant (age at the time of starting the treatment), age of the mother and father, their level of education, their age, the type of oro-facial congenital anomaly, and the order of the affected infant within the family. The responses of mothers evaluating the possible risk factors for CLP were reported. No data regarding smoking were recorded due to the cultural profile of the mothers. The assessment of mothers’ perception and experience regarding PSIO was executed using 16 validated preformed questions. The onset of the sign of the positive correction of the cleft segments was recorded accordingly. In addition, PSIO management, including the removable appliance’s hygiene maintenance, was also assessed.

Overall, 118 infants were enrolled as their parents fully completed survey. Parents were invited to participate in the study after the application of the PSIO for a period of 3 months. The purpose of using PSIO was to approximate and align the palatal segments to facilitate the surgical closure of the lip. The parents were informed and taught how to insert, remove, and activate the appliance. Checking and the adjustment of the PSIO were performed every two weeks until the completion of the procedure. The babies were referred to a plastic surgeon to perform the lip repair at 3–6 months of age. The collection of responses was carried out by an independent co-worker to ensure the double-blindness of the data collection. A valid, informed consent form was obtained from all caregivers. Statistical analysis was carried out using statistical package for social sciences (SPSS) version 23 and Microsoft Office Excel 2010. Quantitative data were presented as number and percentage, whereas quantitative variables were expressed as mean, standard deviation, and range. Difference in mean was assessed using a Student’s t-test (age of the cleft babies). Association between qualitative variables was assessed using chi-squared test. The level of statistical significance was considered at *p* ≤ 0.05 (*).

## 3. Results

The basic characteristics of the involved infants with diagnosed CLP are presented in Table 1. The mean age was 3.02 years ± 0.96 months. Male infants accounted for 68 (57.6%) and the female gender—50 (42.4%). According to CLP classification and severity, infants were distributed as 5.1% (cleft and lip alveolus) vs. 35.6% (unilateral CLP) vs. 59.3% (bilateral CLP). The order of affected children within groups of siblings was mostly between the first and third, and most families admitted to having a single child affected by a CLP anomaly. There was no significant association between any of these characteristics and the mother’s working status/occupation (*p* > 0.05).

Most of the enrolled fathers and mothers were above 20 years of age; however, a significant proportion of mothers (16.9%) were under the age of 20 years (Table 2). Considering the level of education, most of the parents completed their primary education only, and those with higher education (university curriculum) accounted for less than 20% of cases. There was no significant association between any of these characteristics and the mother’s occupation (*p* > 0.05).

### 3.1. The Evaluation of Risk Factors for a Cleft Lip and Palate

The results of evaluating the possible risk factors for CLP are shown in Table 3. Consanguinity was reported by 66.1% of participants and pregnancy was not planned in 83.1% of respondents. Bottled water was the main source of drinking water as reported by 96.6% of participants. Vitamin and mineral intake were subsequently reported in 78.0% of cases. Threatened abortion was reported in 13.6% cases and morning sickness was recorded by 49.2% of mothers. Pharmacotherapy was noted in 39.0% of cases. Severe psychological problems were detected in 16.9% respondents. A balanced, appropriate food intake was observed in 71.2%, while paternal smoking was reported by more than half of the mothers (59.3%). Previous abortions were reported in 39.0%. There was no significant association between any of these potential, hypothetical risk factors and the mother’s occupation or working status (*p* > 0.05).

### 3.2. The Perception and Experience Associated with PSIO Use

The assessment of the mothers’ perception and experience with respect to PSIO is shown in Table 4. The mothers’ concern regarding the impression procedure was reported by a minority (6.8%) and it was significantly associated with housewife status, in comparison with being an employee (*p* = 0.019). About 12% of mothers reported that their infant might suffer from an impression procedure, and this belief was also significantly associated with working status (*p* = 0.009). Physical trauma, whether of an intra- or extra-oral origin, was not reported. Almost all mothers considered the impression as being non-invasive (96.6%), whereas 62% of them reported no difficulties in following the instructions provided by the orthodontist, regarding the insertion of the PSIO and/or taping the elastic band. All respondents were convinced that CLP infants always require PSIO treatment; however, most of the mothers (94.9%) did not search the Internet for information about PSIO therapy. Overall, mothers agreed that PSIO treatment is useful for your infants, improves the infant’s feeding, improves the esthetic of the infant’s facial appearance and facilitates earlier surgical repair. What is more, all respondents were satisfied with the effectiveness of PSIO, equally encouraging other parents to consent for PSIO treatment. They sincerely believed that the orthodontist’s involvement and multidisciplinary approach comprised an important aspect of CLP management. The response to the last 14 questions showed no significant association with the mothers’ occupation (*p* > 0.05).

### 3.3. The Overall PSIO Satisfaction and Orthodontic Appliance Hygiene Regime

Almost half of the respondents (45.8%) noticed a positive correction in the two segments after four months (Table 5) and the results were not significantly associated with the mother’s occupation (*p* = 0.152). Two thirds of the mothers (64.4%) tended to remove the appliance twice a day for cleaning (Table 6), regardless of the mother’s occupation (*p* = 0.219). Most of the respondents (79.7%) admitted that the orthodontic approach was successful in the treatment (Table 7, *p* = 0.763).

## 4. Discussion

The research hypothesis was tested to determine comparatively discrepancy in knowledge of mothers of children with CLP regarding attitude towards PSIO, considering their occupational status. The treatment outcome of children with CLP, their parents’ quality of life, and ongoing suffering are determined by the severity of anatomic disfigurement, functional outcomes regarding feeding and speech, as well as emotional and psychological impacts. Therefore, the surgical correction of the anatomical anomaly is undoubtedly the ultimate means by which these adverse outcomes can be reduced and managed successfully. In most instances, such a surgical correction is not amenable during the early months of the infant’s life, extending the time before definite corrective surgery is accomplished. As a result, a transitory period between the delivery and time of surgical correction is associated with considerable adverse functional and psychological disturbances that require medical intervention. It is assumed that the use of PSIO significantly reduces functional and psychological burdens and will offer a smooth transitory period until the time of the operation. In addition, it has been revealed that PSIO is associated with a narrowing of the defects and an approximation of its margins, allowing the alignment of the palatal segments, so that the outcome of the definitive surgical correction is optimized and enhanced.

Due to the relatively sparse existing evidence, this cross-sectional study was carried out to determine the attitudes, perceptions, and experience of mothers of children with CLP undergoing a PSIO application for 3 months prior to definitive surgical correction. Notably, a negative perception concerning the PSIO was reported in a minority of participants (6.8%) and a lower rate of infants suffering from such appliances based on the mothers’ experiences, as only 11.9% reported such suffering, and these concerns were significantly more associated with being a housewife than being employed. Interestingly, the employed mothers revealed less knowledge regarding the impression procedure, compared to PSIO use overall, which indicates the need to appropriately educate parents looking after children with CLP. The possible explanation for this observation is that employed mothers are more educated and have better access to sources of knowledge concerning the advantages of PSIO for their children. Therefore, the employed mothers are deemed to be less afraid of the disadvantages and adverse effects of these appliances. Whilst a fraction of the surveyed mothers reported their concern associated with the traditional impression procedure, orthodontists should consider using alternative modern methods, such as 3D digital impressions utilizing intraoral scanners or creating a guide with photos, and teaching the parents step by step how to handle this appliance.

Consistently, evidence of physical trauma attributed to the PSIO was not reported by respondents. Moreover, the mothers were able to follow the post-operative instructions and handle the appliances, and most of them reported no difficulties in handling the appliance. Conversely, 38% of the mothers reported a difficulty in handling this type of appliance and mentioned their burden when it comes to activating the appliance and attending the visits. Thus, considering other types of appliances (passive appliance) or substitute by lip taping can be considered in families who have such difficulties. At least, the majority expressed that the PSIO treatment is useful for the infant and improves the infant’s feeding, including the aesthetics of the infant’s facial appearance and enabling the surgical repair promptly. Moreover, all mothers were satisfied with the outcomes of PSIO, subsequently encouraging other parents to consent to the PSIO treatment and were convinced that the orthodontist’s support is an important part of the CLP team.

Despite the PSIO’s benefits, substantial controversy still exists associated with the treatment of children with CLP worldwide [4]. Both parents and health care workers find difficult and challenging dealing with infants with CLP during their first weeks of life [39]. Previous studies evaluated parents’ opinions related to the use of PSIO. Primarily, the outcome of definite surgical interventions has been assessed in randomized controlled clinical trials in which the prior use of PSIO versus no use was assessed [4,40]. A cross-sectional study executed by Prahl et al. compared mothers’ attitude towards the CLP management of their infants in two cohorts with and without appliance use, and no significant difference was reported between these two groups [23]. In this study, data about the disadvantage of using moldings were not clearly defined or discussed; therefore, it is difficult to compare the results. According to Sischo et al., the majority of caregivers experienced initial uncertainty and apprehension about the burden of care associated with moldings, e.g., cleaning the appliance and changing and positioning the tape [29]. In later interviews, however, caregivers usually reported a positive perception with respect to their active participation in their child’s CLP management. These positive attitudes were correlated with feelings of empowerment and increased self-esteem. Nevertheless, parents reported some reluctance associated with the use of PSIO moldings; however they tried their best to reduce the impact of PSIO discomfort in comparison with the perceived long-term benefits.

Overall, the results of this aforementioned study [29] are in line with the findings of our survey, which is also supported by the results of another study [30], concluding that the use of molding greatly reduced the psychological burden and greatly improved functional outcomes in infants with CLP. In a study conducted by Broder et al. in high-volume cleft centers [31], ’molding’ caregivers reported better post-surgery outcomes compared with the no-molding CLP infants’ caregivers (*p* < 0.05), particularly in relation to the appearance of the nose. These results are supportive and in line with our findings. Similarly, Hopkins et al. [32], who explored the experience of parents caring for an infant with a CLP molding, observed that parents were committed to the treatment process and believed that the benefits exceeded the additional work that nasoalveolar molding requires, which is in accordance with our findings.

### 4.1. Strengths and Limitations of the Study

A single-center study limited to one country may prevent the wide extrapolation of the obtained results. However, such a study design seems sufficient to provide reliable and robust data within the regional level. Even though the sample size allowed for the sound validation of the quantitative results, a multi-center international research project, involving variable groups of subjects, might further enhance the data reliability. Contrarily, the country-specific differences in PSIO perception by care givers can considerably affect the null hypothesis about parents’ attitudes towards PSIO use. The lack of standardized formulae containing the satisfaction survey designed for parents who look after their children with CLP restricts the transferability of the results. Further surveys are required to verify the clinical usefulness of PSIO. The local healthcare systems organization and national health services could vastly contribute to PSIO arrangement.

### 4.2. Implications

The obtained results may have a substantial influence in public health strategies targeting patients with CLP, as well as being able to provide evidence-based data for authorities’ policy related to the healthcare management of CLP cases. The wider and earlier implementation of PSIO may positively change therapeutic protocols worldwide and improve therapeutic outcomes. Local health boards and medical teams are required to rely on the most current and validated study results when dealing with CLP patients. The promotion of early CLP interventions focusing on a wide range of populations should be the ultimate goal of protocols and new national guidelines.

## 5. Conclusions

The mothers of infants with CLP, regardless of their occupational status, have a positive attitude towards PSIO, expressing only minor concerns. They acknowledge its clinical importance as the application of PSIO leads to feeding improvement, normalization of speech, as well as better definitive surgical outcomes. Parents should be aware of the usefulness of PSIO, which can significantly improve the clinical outcome, prognosis, and CLP infants’ quality of life.

## Figures and Tables

**Table 1:** The primary characteristics of infants diagnosed with CLP, with a reference to their mothers.

Characteristic	Total *n* = 118	Housewife *n* = 72	Employee *n* = 46	*p*
**Age (months)**				
Mean ± SD	3.02 ± 0.96	1.99 ± 0.97	2.07 ± 0.93	0.626
Range	1–4	1–4	1–4	
**Gender**				
Male, *n* (%)	68 (57.6%)	44 (61.1%)	24 (52.2%)	0.338
Female, *n* (%)	50 (42.4%)	28 (38.9%)	22 (47.8%)	
**Cleft Severity**				
Cleft lip, *n* (%)	0 (0.0%)	0 (0.0%)	0 (0.0%)	0.642
Cleft palate, *n* (%)	0 (0.0%)	0 (0.0%)	0 (0.0%)	
Cleft lip and alveolus, *n* (%)	6 (5.1%)	3 (4.2%)	3 (6.5%)	
Unilateral CLP, *n* (%)	42 (35.6%)	24 (33.3%)	18 (39.1%)	
Bilateral CLP, *n* (%)	70 (59.3%)	45 (62.5%)	25 (54.3%)	
**Patients Order Among Siblings**				
First, *n* (%)	24 (20.3%)	14 (19.4%)	10 (21.7%)	0.510
Second, *n* (%)	42 (35.6%)	25 (34.7%)	17 (37.0%)	
Third, *n* (%)	26 (22.0%)	16 (22.2%)	10 (21.7%)	
Fourth, *n* (%)	18 (15.3%)	10 (13.9%)	8 (17.4%)	
Fifth, *n* (%)	6 (5.1%)	6 (8.3%)	0 (0.0%)	
Sixth, *n* (%)	2 (1.7%)	1 (1.4%)	1 (2.2%)	
**Patients Order Among Affected**				
First, *n* (%)	112 (94.9%)	68 (94.4%)	44 (95.7%)	0.771
Second, *n* (%)	6 (5.1%)	4 (5.6%)	2 (4.3%)	

*n*: number of cases; SD: standard deviation; CLP: cleft lip and palate.

**Table 2:** The characteristics of care givers of CLP infants.

Characteristic	Total *n* = 178	Housewife *n* = 72	Employee *n* = 46	*p*
	*n* (%)	*n* (%)	*n* (%)	
**Father’s Age (years)**				
<20	2 (1.7%)	1 (1.4%)	1 (2.2%)	0.949
20–30	62 (52.5%)	38 (52.8%)	24 (52.2%)	
30–40	54 (45.8%)	33 (45.8%)	21 (45.7%)	
**Mother’s Age (years)**				
<20	20 (16.9%)	12 (16.7%)	8 (17.4%)	0.745
20–30	76 (64.4%)	45 (62.5%)	31 (67.4%)	
30–40	22 (18.6%)	15 (20.8%)	7 (15.2%)	
**Father’s Education**				
Illiterate	2 (1.7%)	1 (1.4%)	1 (2.2%)	0.925
Primary	70 (59.3%)	43 (59.7%)	27 (58.7%)	
Intermediate	14 (11.9%)	9 (12.5%)	5 (10.9%)	
High school	10 (8.5%)	7 (9.7%)	3 (6.5%)	
University	22 (18.6%)	12 (16.7%)	10 (21.7%)	
**Mother’s Education**				
Illiterate	12 (10.2%)	7 (9.7%)	5 (10.9%)	0.999
Primary	60 (50.8%)	37 (51.4%)	23 (50.0%)	
Intermediate	24 (20.3%)	15 (20.8%)	9 (19.6%)	
High school	10 (8.5%)	6 (8.3%)	4 (8.7%)	
University	12 (10.2%)	7 (9.7%)	5 (10.9%)	

*n*: number of cases; CLP: cleft lip and palate.

**Table 3:** Results addressing the risk factors for cleft lip and palate.

Questions	Housewife *n* = 72				Employee *n* = 46				*p*
	No		Yes		No		Yes		
	*n*	%	*n*	%	*n*	%	*n*	%	
Are the parents relative?	25	34.7	47	65.3	15	32.6	31	67.4	0.813
Was the pregnancy planned?	61	84.7	11	15.3	37	80.4	9	19.6	0.545
Was bottled water the source of drinking water?	4	5.6	68	94.4	0	0.0	46	100.0	0.104
Did mother take a vitamins and/or folic acid during this pregnancy?	19	26.4	53	73.6	7	15.2	39	84.8	0.153
Did mother have a history of threatened abortion?	64	88.9	8	11.1	38	82.6	8	17.4	0.331
Did mother suffer from morning sickness?	36	50.0	36	50.0	24	52.2	22	47.8	0.818
Did mother have a history of drug intake?	45	62.5	27	37.5	27	58.7	19	41.3	0.679
Did mother have a history of severe psychological problems?	57	79.2	15	20.8	41	89.1	5	10.9	0.159
Did mother have a good food intake?	25	34.7	47	65.3	9	19.6	37	80.4	0.076
Did father smoke before and during pregnancy?	28	38.9	44	61.1	20	43.5	26	56.5	0.621
History of previous abortions?	42	58.3	30	41.7	30	65.2	16	34.8	0.455

**Table 4:** Mother’s perception and experience related to PSIO in association with working status.

Characteristic	Housewife *n* = 72				Employee *n* = 46				*p*
	No		Yes		No		Yes		
	*n*	%	*n*	%	*n*	%	*n*	%	
How often have you been worried after the explanation of the impression-taking procedure?	64	88.9	8	11.1	46	100.0	0	0.0	0.019 *
Do you think your infant suffered during the impression method?	59	81.9	13	18.1	45	97.8	1	2.2	0.009 *
Have you found any sign of intra- or extra-oral physical trauma after the impression procedure?	72	100.0	0	0.0	46	100.0	0	0.0	--
How invasive was the method of impression taking?	68	94.4	4	5.6	46	100.0	0	0.0	0.104
Do you face any difficulties in following the instructions of the orthodontist, in regard to the insertion of the PSIO and/or taping the elastic band?	43	59.7	29	40.3	31	67.4	15	32.6	0.401
“Do you think that CLP infants always need PSIO treatment?”	0	0.0	72	100.0	0	0.0	46	100.0	--
“Did you search the web looking for information about PSIO treatment?”	68	94.4	4	5.6	44	95.7	2	4.3	0.771
“Did you find PSIO treatment useful for your infant?”	0	0.0	72	100.0	0	0.0	46	100.0	--
“Did it improve the infant’s feeding?”	0	0.0	72	100.0	0	0.0	46	100.0	
“Did it improve the esthetic of the infant’s facial appearance, in regard to lip, nose, and profile of the face?”	4	5.6	68	94.4	0	0.0	46	100.0	0.104
“Did it make the appointment for surgical repair earlier?”	0	0.0	72	100.0	0	0.0	46	100.0	--
“Did you find it embarrassing that your infant was wearing the appliance?”	72	100.0	0	0.0	46	100.0	0	0.0	--
“How satisfied are you with the outcomes of the PSIO?”	0	0.0	72	100.0	0	0.0	46	100.0	--
“Would you encourage other parents to do the PSIO treatment?”	0	0.0	72	100.0	0	0.0	46	100.0	--
“Do you think that the orthodontist is an important member of the cleft team?”	0	0.0	72	100.0	0	0.0	46	100.0	--
“To what extent has the cleft influenced your family’s life?”	19	26.4	53	73.6	11	23.9	35	76.1	0.763

*n*: number of cases; PSIO: pre-surgical infant orthopedic; CLP: cleft lip and palate. *—statistically significant.

**Table 5:** Responses to the question: “When did you notice the positive correction of the cleft segments?” in association with working status.

Week	Housewife *n* = 72		Employee *n* = 46		χ^2^	DF	*p*
	*n*	%	*n*	%			
One	29	40.3	25	54.3			
Two	25	34.7	17	37.0	5.281	3	0.152
Three	8	11.1	2	4.3			
Four	10	13.9	2	4.3			

*n*: number of cases.

**Table 6:** Mother’s responses to the question: “How many times did you remove the appliance from your baby’s mouth for cleaning?” in association with working status.

Count	Housewife *n* = 72		Employee *n* = 46		χ^2^	DF	*p*
	*n*	%	*n*	%			
1	5	6.9	3	6.5			
2	43	59.7	33	71.7			
3	16	22.2	10	21.7	5.751	4	0.219
4	6	8.3	0	0.0			
6	2	2.8	0	0.0			

*n*: number of cases; DF: degree of freedom.

**Table 7:** Study results referred to the question: “How successful do you think that the orthodontists been in your treatment?” taking into consideration working status.

Response	Housewife *n* = 72		Employee *n* = 46		χ^2^	DF	*p*
	*n*	%	*n*	%			
None	0	0	0	0			
Not much	0	0	0	0			
Little	0	0	0	0			
A lot	14	19.4	10	21.7	0.091	1	0.763
Excellent	58	80.6	36	78.3			

*n*: number of cases; DF: degree of freedom.

## Data Availability

Raw data available on request.
